# Promoter analysis of macrophage- and tick cell-specific differentially expressed *Ehrlichia chaffeensis *p28-Omp genes

**DOI:** 10.1186/1471-2180-9-99

**Published:** 2009-05-19

**Authors:** Lalitha Peddireddi, Chuanmin Cheng, Roman R Ganta

**Affiliations:** 1 Department of Diagnostic Medicine/Pathobiology, College of Veterinary Medicine, Kansas State University, Manhattan, KS 66506, USA

## Abstract

**Background:**

*Ehrlichia chaffeensis *is a rickettsial agent responsible for an emerging tick-borne illness, human monocytic ehrlichiosis. Recently, we reported that *E. chaffeensis *protein expression is influenced by macrophage and tick cell environments. We also demonstrated that host response differs considerably for macrophage and tick cell-derived bacteria with delayed clearance of the pathogen originating from tick cells.

**Results:**

In this study, we mapped differences in the promoter regions of two genes of p28-Omp locus, genes 14 and 19, whose expression is influenced by macrophage and tick cell environments. Primer extension and quantitative RT-PCR analysis were performed to map transcription start sites and to demonstrate that *E. chaffeensis *regulates transcription in a host cell-specific manner. Promoter regions of genes 14 and 19 were evaluated to map differences in gene expression and to locate RNA polymerase binding sites.

**Conclusion:**

RNA analysis and promoter deletion analysis aided in identifying differences in transcription, DNA sequences that influenced promoter activity and RNA polymerase binding regions. This is the first description of a transcriptional machinery of *E. chaffeensis*. In the absence of available genetic manipulation systems, the promoter analysis described in this study can serve as a novel molecular tool for mapping the molecular basis for gene expression differences in *E. chaffeensis *and other related pathogens belonging to the *Anaplasmataceae *family.

## Background

*Ehrlichia chaffeensis*, an obligate, intracellular, tick-borne bacterium that belongs to the family *Anaplasmataceae*, is responsible for an emerging disease in humans called human monocytic ehrlichiosis (HME) [1,2]. The transmitting vector of *E. chaffeensis, Amblyomma americanum*, acquires the pathogen during a blood meal from an infected host [2]. Host cell adaptation and establishment of persistent infection in tick and vertebrate hosts are critical for successful completion of the *E. chaffeensis *lifecycle and, similarly, for other tick-transmitted rickettsiales of the genera *Ehrlichia *and *Anaplasma *[3-7]. It is necessary for the tick-transmitted pathogens to have evolved strategies that support host cell adaptation and to establish persistent infections. There may be many ways by which the pathogens persist; strategies may include altering the host response [8,9], varying expressed proteins relative to time post-infection and differential host-specific protein expression [10-19].

Recently, we reported that *Ehrlichia *species alter the expression of many proteins in a host cell-specific manner [18-21]. Differentially expressed proteins include outer membrane proteins made from p28-Omp multigene locus having 22 tandomly arranged paralogous genes of *E. chaffeensis *[18-20]. The major expression from this locus is limited to a subset of genes and is also influenced by vertebrate and tick cell environment. P28-Omp 14 protein is the major expressed protein when *E. chaffeensis *is grown in tick cells, whereas p28-Omp 19 is expressed predominantly by the organism in macrophages. We also reported that the pathogen originating from tick cells persists longer in a vertebrate host and the host response is significantly different for tick cell-derived bacteria compared with bacteria originating from macrophages [9].

Little is known about the promoter structures and transcriptional regulation of *E. chaffeensis *genes and their contributions to alter the gene expression in response to tick and vertebrate host cell environments. Promoter analysis under *in vivo *conditions is not possible at this time because of a lack of methods to transform *E. chaffeensis*. In the current study, we report the first description of mapping promoter regions of two host-specific differentially expressed genes of *E. chaffeensis*.

## Results

### Primer extension analysis of p28-Omp genes 14 and 19

**Figure 1:**
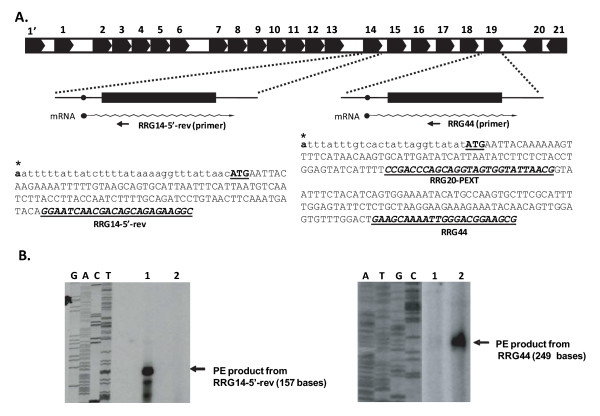
**Primer extension (PE) analysis of p28-Omp genes 14 and 19**. Panel A has a cartoon spanning all 22 genes [37]. This panel also has an expansion of cartoons for genes 14 and 19 with predicted transcripts, the primers used for the PE analysis and sequences of the primer extended products with transcription start sites identified with asterisks. PE analysis products resolved on a sequencing gel are shown in panel B. Blots on the left and right represent the data for transcripts of genes 14 and 19, respectively. A sequence ladder for the gene 14 analysis was prepared by using the same primer used for the PE analysis but with a DNA template spanning the gene 14 sequence. For gene 19, PE analysis was performed with RRG 44 primer, and the sequencing ladder was generated by using RRG20-PEXT primer with a gene 19 DNA template. (Lane 1, *E. chaffeensis *RNA from tick cells; lane 2, *E. chaffeensis *RNA from macrophages).

Host-specific differential protein expression from numerous *E. chaffeensis *genes, including from p28-Omp multi-gene locus, has been reported previously [18-20]. To evaluate the gene expression at transcription level, primer extension analysis was performed for p28-Omp genes 14 and 19 with macrophage and tick cell-derived *E. chaffeensis *RNA (Figure 1A and 1B). The primer extended products for genes 14 and 19 were detected in tick cell- and macrophage-derived *E. chaffeensis *RNA, respectively (Figure 1). The analysis also aided in identifying the transcription start sites for genes 14 and 19 located at 34 and 26 nucleotides upstream to the initiation codons, respectively (Figure 1). The nucleotide at the transcription start sites for both the genes is adenosine.

### Transcriptional analysis by quantitative RT-PCR at different times post-infection

**Figure 2:**
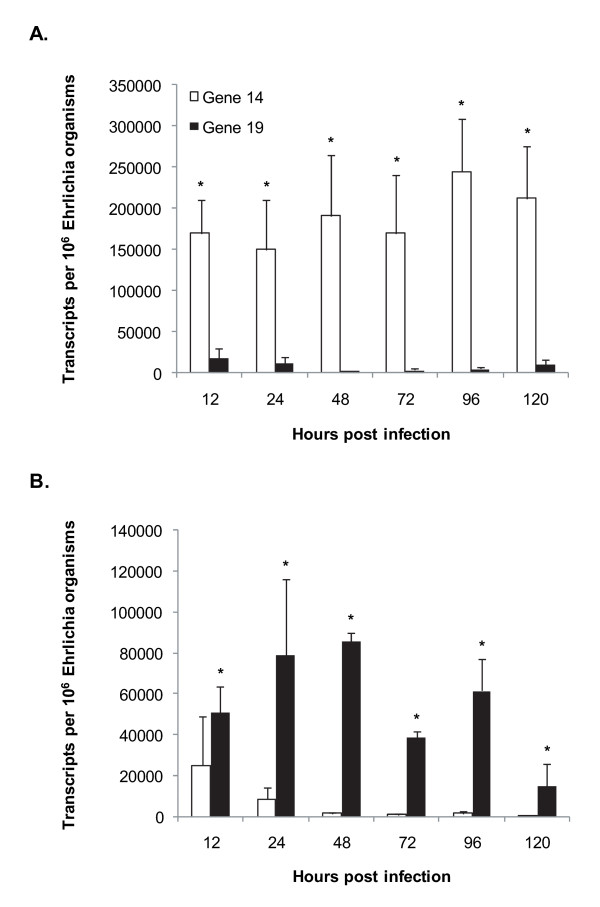
**Quantitative RT-PCR analysis**. TaqMan-based quantitative RT-PCR analysis was performed with RNA isolated from tick cell (A) and macrophage (B) cultures harvested at different times postinfection. Transcript numbers were estimated and presented per million *E. chaffeensis *organisms. Data are presented with SE values calculated from three independent experiments (*P *≤ 0.05).

Our previous studies suggested that both p28-Omp genes 14 and 19 are transcriptionally active in *E. chaffeensis *originating from vertebrate macrophages and tick cells but the expression levels are different [9,19]. The quantitative gene expression differences for genes 14 and 19 were determined by TaqMan-based real-time RT-PCR analysis (quantitative RT-PCR) (Figure 2). Consistent with the previous observations, transcripts for genes 14 and 19 were detected in RNA isolated from both host cell backgrounds. In tick cell-derived *E. chaffeensis*, p28-Omp gene 14 expression remained higher than expression of p28-Omp gene 19 (Figure 2A). The gene 14 expression in *E. chaffeensis *also remained high for all time points analyzed post-inoculation in tick cells. In macrophage-derived *E. chaffeensis*, expression levels were reversed with significantly higher expression for gene 19 (Figure 2B).

### P28-Omp 14 and 19 promoter regions sequence analysis

**Figure 3:**
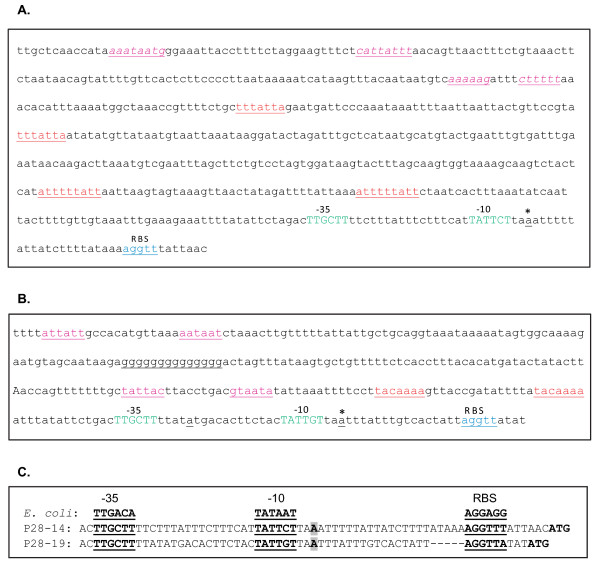
**P28-Omp genes 14 and 19 promoter region sequence analysis**. Upstream sequences of genes 14 (panel A) and 19 (panel B) were evaluated for the presence of direct repeats (red text), palindromic sequences (pink text) and for the presence of unique sequences (G-rich region), consensus -35 and -10 regions (green text) and ribosome binding sites (blue text). Panel C shows the comparison of -10, -35 and ribosome binding sites of genes 14 and 19 with the *E. coli *consensus sequences. Transcription start sites for the genes mapped by primer extension analysis are identified with bold and grey color highlighted text or with an asterisk. Dashes were introduced in the p28-Omp gene 19 sequence to create alignment with the gene 14 sequence.

The entire non-coding sequences upstream to genes 14 and 19 were evaluated to identify sequences similar to the consensus *E. coli *RNA polymerase binding site sequences, -10 and -35, and ribosome binding site sequences (RBS) (Figure 3). Consensus -10 and -35 elements were identified and are located few bases upstream to the transcription start sites mapped by primer extension analysis (Figure 3). Similarly, putative RBS sequences [22] were identified 7 and 4 nucleotides upstream to the initiation codon of genes 14 and 19, respectively. Genes 14 and 19 sequences upstream to the predicted -10 and -35 sequences differed considerably in their lengths and homology (Figure 3A and 3B). The gene 14 upstream sequence is 581 bp in length, which is 273 bp longer than the gene 19 upstream sequence (308 bp). The sequences included several gene-specific direct repeats and palindrome sequences. In addition, a unique 14 nucleotide-long 'G' rich sequence was detected in the gene 19 sequence. The consensus -35 sequence was identical for both the genes, whereas the -10 and RBS sequences differed by one nucleotide each (Figure 3C). Relative distances of the consensus -10 and -35 sequences from transcription start sites also remained the same for both the genes (Figure 3C).

### Evaluation of promoter activities of the sequences upstream to the coding regions of the p28-Omp genes 14 and 19

The transcription analysis assessed by direct RNA mapping and TaqMan-based RT-PCR methods revealed quantitative differences in gene expression for p28-Omp genes 14 and 19, which is influenced by invertebrate and vertebrate host cell environments. It is unclear how the host cell environments influence the *Ehrlichia *gene expression. Promoter analysis of these differentially expressed genes will be valuable for gaining insights about how differential expression is achieved by *E. chaffeensis *in vertebrate and tick host environments. Promoter characterization *in vivo *for *E. chaffeensis *is not feasible at this time because genetic manipulation systems are yet to be established. Alternatively, characterization of *E. chaffeensis *promoters may be performed in *E. coli *or with *E. coli *RNA polymerase as reported for several *C. trachomatis *genes [23-30].

**Figure 4:**
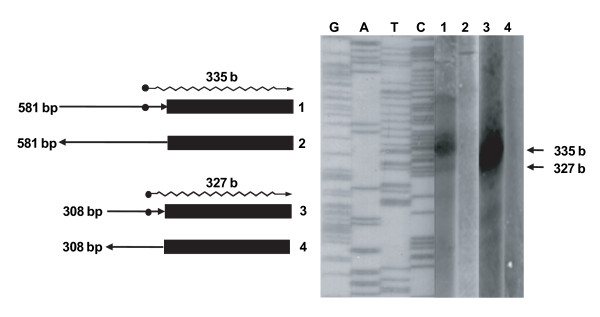
***In vitro *transcription analysis**. *In vitro *transcription analysis was performed for the complete upstream sequences of genes 14 and 19 in forward and reverse orientations ligated to a partial lacZ gene segment (301 bp) (solid black boxes). The orientation of ligated promoter regions is shown by arrowhead lines (right arrowhead line, forward orientation; left arrowhead line, reverse orientation). Wiggled arrowhead lines show predicted transcripts of 335 bases for gene 14 and 327 bases for gene 19. Sequence segments and the predicted transcripts for genes 14 and 19 are shown as cartoons on the left, and the observed transcripts are shown on the right of the panels. Puc18 plasmid DNA was used as the template to generate a sequence ladder with an M13 forward primer. Numbers 1 and 2 refer to the constructs for *in vitro *transcription for gene 14, and 3 and 4 refer to *in vitro *transcription templates for gene 19.

To validate the use of *E. coli *for mapping the promoters of *E. chaffeensis *genes,*in vitro *transcription assays were performed for p28-Omp 14 and 19 promoter regions with *E. coli *RNA polymerase by following methods reported for *Chlamydia *species [28-30]. Predicted *in vitro *transcripts, as estimated from transcription start sites mapped by primer extension described previously, were detected only when p28-Omp 14 and 19 complete upstream sequences were ligated to a segment of lacZ coding sequence (Figure 4). *In vitro *transcripts were absent in the reactions that contained the complete gene 14 and 19 promoter regions ligated in reverse orientation (Figure 4).

**Figure 5:**
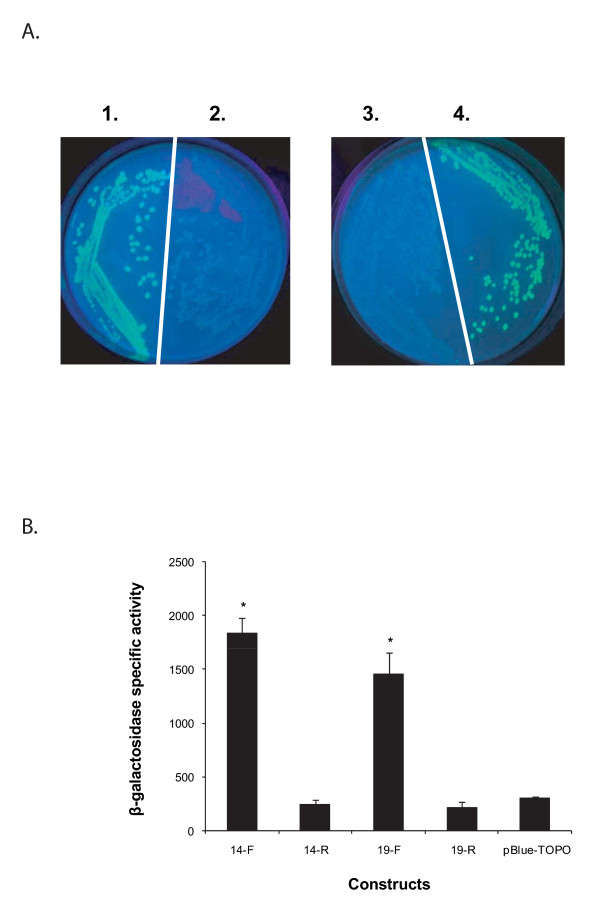
**(A) Green fluorescent protein (GFP) constructs evaluated for the promoter activity of p28-Omp genes 14 and 19**. The pPROBE-NT plasmids containing the promoterless GFP gene (2 and 3) and upstream sequences of genes 14 and 19 in front of the GFP gene (1 and 4, respectively) and a construct containing no promoter sequence were evaluated for GFP expression in *E. coli*. (B) LacZ constructs evaluated for the promoter activity of p28-Omp genes 14 and 19. The pBlue-TOPO vector containing promoterless lacZ gene (pBlue-TOPO) and upstream sequences of genes 14 and 19 inserted in forward (14-F and 19-F) and reverse orientations (14-R and 19-R) were evaluated for β-galactosidase activity in *E. coli*. Data are presented with SD values calculated from four independent experiments (*P *≤ 0.001).

Upstream sequences for p28-Omp genes 14 or 19 were subsequently evaluated in *E. coli*. Transformants of *E. coli *containing promoter regions of genes 14 and 19 cloned in front of the promoterless green fluorescent protein (GFP) coding sequence in the pPROBE-NT plasmid were positive for green fluorescence as visualized by the presence of green color colonies (Figure 5A). *E. coli *transformed with pPROBE-NT plasmids alone were negative for the green fluorescence. The GFP expression was verified by Western blot analysis with GFP-specific polyclonal sera (not shown). Promoter activities for upstream sequences of genes 14 and 19 were further confirmed by another independent method (i.e., by assessing the β-galactosidase activity after inserting the sequences in front of the promoterless lacZ gene in pBlue-TOPO plasmid). The *E. coli *transformants with plasmids having gene 14 or 19 sequences cloned in correct orientation had significantly more β-galactosidase activity (*P *≤ 0.001) than the baseline activity observed for constructs with no promoter sequences or when the sequences were inserted in reverse orientation (Figure 5B).

### Promoter deletion analysis

**Figure 6:**
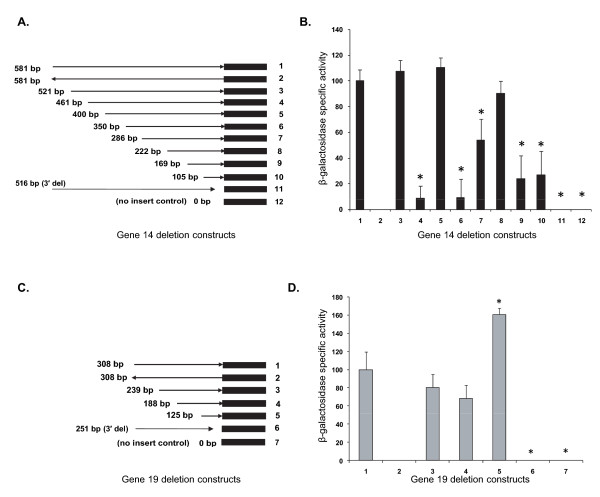
**Deletion analysis of promoter regions of genes 14 and 19**. β-galactosidase activity of extracts prepared from *E. coli *cultures of bacteria transformed with various deletion constructs was determined. Panels A and C have cartoons depicting deletion constructs and their orientations for genes 14 and 19, respectively. (Solid black boxes represent lacZ gene, and right and left arrowhead lines show orientation of the promoter regions ligated in front of the lacZ coding sequence. Lengths of the promoter regions in base pairs are indicated on the left. Panels B and D contain the β-galactosidase activity analysis data. (β-galactosidase activity was expressed as percent activity relative to the activity observed for full length promoter segments.) Data are presented with SD values calculated from four independent experiments (*P *≤ 0.001).

Deletion analyses were performed to assess whether the promoter activities are influenced by the sequences upstream to the transcription start sites of genes 14 and 19; β-galactosidase activity for several pBlue-TOPO plasmid constructs with segments deleted from the 5' end for both the genes were evaluated (Figure 6). Deletions to the sequences ranged from 60 to 476 bp for p28-Omp gene 14 and 69 to 183 bp for gene 19. All deletion constructs for gene 14, except for deletions having 461 and 350 bp segments, had significantly higher β-galactosidase activity compared with negative controls lacking no insert and the insert in the reverse orientation. The first 60 bp deletion from the 5' end resulted in no significant change in β-galactosidase activity compared with that observed for the full-length insert, whereas a deletion of an additional 60 bp caused a decline of about 90% of the enzyme activity. The β-galactosidase activity was restored completely by an additional 61 bp deletion. Further deletion of another 50 bp also resulted in another near-complete loss of activity. Subsequent deletions of 64 bp each caused a stepwise restoration of the enzyme activity to 54 and 91%, respectively. Deletion of another 53 bp caused another drop in β-galactosidase activity to 24%, which remained unaffected with an additional deletion of a 64 bp fragment (Figure 6A and 6B). Similar deletion analysis performed for the gene 19 upstream sequence also resulted in altered β-galactosidase activity compared with the full-length sequence (Figure 6, panels C and D). The 5' end deletions of 69 and 120 bp for this gene resulted in a 20 and 30% decline, respectively, in enzyme activity. These declines, however, were not statistically significant. Deletion of an additional 63 bp caused an increase of about 60% more β-galactosidase activity. To confirm that the RNA polymerase binding regions are located within the sequences spanning up to the consensus -35 sequences, 3' end deletion constructs lacking sequences up to the -35 region for genes 14 and 19 (65 and 57 bp, respectively) were prepared and assessed for β-galactosidase activity. These deletions led to the complete loss of β-galactosidase activity (Figure 6A–B lane 11 and 6C–D lane 6).

### Location of -10 and -35 regions

**Figure 7:**
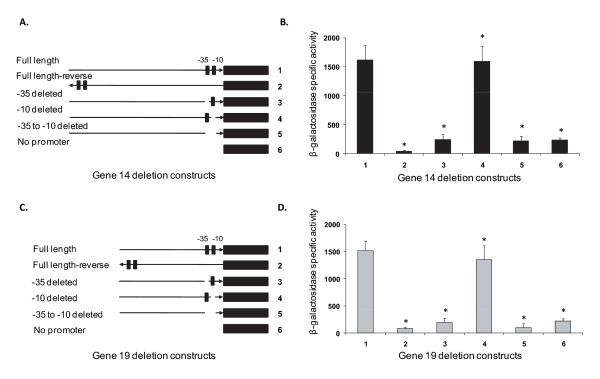
**Deletion analysis spanning the -35 and -10 regions of genes 14 and 19**. β-galactosidase activity of extracts prepared from *E. coli *cultures of bacteria transformed with -35 or -10 deletions or deletions spanning from -35 to 10 were determined. Panels A and C have cartoons depicting deletion constructs and their orientations for genes 14 and 19, respectively. Panels B and D contained the β-galactosidase activity analysis data. Data are presented with SD values calculated from four independent experiments (*P *≤ 0.001).

To determine whether the consensus -35 and -10 represented true RNA polymerase binding site regions, constructs lacking either the predicted -35 or -10 alone or the regions spanning from -35 to -10 were generated, and the effect of the loss of these sequences on promoter activity was evaluated by measuring β-galactosidase activity. Deletion of the predicted -35 regions alone or in combination with the -10 for both the genes resulted in decline of β-galactosidase activity to the background levels observed for negative controls. Deletion of the consensus -10 region alone for both the genes, however, resulted in no significant change to the promoter activity (Figure 7). The impact of the deletions of -35 and -10 are very similar for both genes' promoters.

## Discussion

Differences in protein expression influenced by vertebrate and tick cell environment are now well documented for *E. chaffeensis *[18-20] and other tick-transmitted bacteria [12,13,15,16]. We recently reported novel data describing differences in immune response in the murine host against *E. chaffeensis *originating from tick cells compared with that observed for the bacteria originating from macrophages [9]. Importantly, the murine host takes longer to clear the pathogen originating from tick cells, and the delayed clearance has been associated with altered macrophage, B-cell and cytokine responses. These studies suggest that tick cell-specific altered pathogen protein expression offers a selective advantage to *E. chaffeensis *for its continued survival when it enters into a vertebrate host from the tick cell environment. To date, no studies have assessed the molecular mechanisms used by *E. chaffeensis *to achieve differential gene expression.

Primer extension analysis reported in this study confirmed our previous observations of Northern blot analysis that transcripts of p28-Omp genes 14 and 19 are differentially expressed and as monocistronic messages [19]. The primer extension analysis also aided in defining transcription start sites. Adenine, the base found at the transcription start site for genes 14 and 19 of *E. chaffeensis*, appears to be the most common base at which transcription is initiated from rickettsiales genes, including pathogens of the genera *Rickettsia *and *Anaplasma *[31-34]. Our previous studies and those of other investigators also support that genes 14 and 19 are transcriptionally active independent of *E. chaffeensis *originating from macrophages or tick cells [9,19,21,35-38]. In the current study, quantitative RT-PCR analysis confirmed the previous observations about the presence of messages for genes 14 and 19 in both host cell backgrounds. In addition, the analysis aided in mapping quantitative differences in transcription of differentially expressed genes. The quantitative RT-PCR analysis demonstrates that although genes 14 and 19 are transcriptionally active, levels of transcription are influenced in response to the macrophage and tick cell environments. Gene 19 is higher in its expression in macrophages, and the opposite is true for gene 14 expression.

Promoter regions of genes 14 and 19 differed considerably; the differences include variations in length of the upstream sequences, presence of several gene-specific direct repeats, palindrome sequences and presence of a G-rich region found in gene 19. Importance of palindrome and direct repeat sequences in regulating transcription is well established for many prokaryotes and for a rickettsial pathogen [34,39-42]. For example, the presence of a palindrome sequence in the citrate synthase gene of *Rickettsia prowazekii *with its possible role in transcriptional regulation is reported by Cai and Winkler [42]. Similarly, transcription factors such as zinc finger proteins that influence gene expression via interacting with G-rich sequences are established for both prokaryotes and eukaryotes [43-49]. The *E. chaffeensis *genome contains two homologs of zinc finger proteins (Genbank #s ABD44730 and ABD45416) [50]. It is of interest to investigate whether one or both of these putative *E. chaffeensis *zinc finger proteins act as transcription regulators for p28-Omp gene 19.

Mapping the functions of *E. chaffeensis *genes *in vivo *cannot be performed because genetic manipulation systems are yet to be established. To overcome this limitation, we assessed the utility of *E. coli *RNA polymerase as a surrogate to characterize *E. chaffeensis *gene promoters as reported for several *C. trachomatis *genes [23-30]. *In vitro *transcription assays performed with *E. coli *RNA polymerase identified the same transcription start sites for p28-Omp genes 14 and 19 as observed in *E. chaffeensis*. This observation validates the use of *E. coli *RNA polymerase.

Molecular characterization of promoter sequences located upstream to the transcription start sites of genes 14 and 19 is critical in determining how *E. chaffeensis *regulates gene expression. In *E. coli*, expression of reporter gene products, GFP and β-galactosidase, is evident when sequences upstream to the coding regions of p28-Omp genes 14 and 19 were placed in front of promoterless GFP or β-galactosidase genes, respectively. These data are also consistent with previous reports that the *E. coli *RNA polymerase can complement the functions of rickettsial RNA polymerases of the genera *Anaplasma, Ehrlichia *and *Rickettsia *[31,32,37], including recognizing the transcription start sites [32].

Sequential deletions in the gene 14 upstream sequences from the 5' end, whereby some of the direct repeats and palindrome sequences were deleted, resulted in variations in the promoter activity that fluctuated from complete or partial loss of activity compared with that observed for the full-length upstream sequence. Additional deletions caused the restoration of 100% activity, and subsequent additional deletions again led to a decline in promoter activity. Similarly, deletion analysis in the gene 19 promoter region caused loss or gain of promoter activities relative to the inclusion of full-length upstream sequence as a promoter. These data suggest that promoter regions of genes 14 and 19 contain sequence domains that influence binding affinity of RNA polymerase to the respective promoters. Altered promoter activities observed in deletion analysis experiments may have resulted from the deletions of upstream sequences involved in altering DNA topology and making RNA polymerase less or more accessible to its binding domains. Influence of 5' sequences altering the DNA topology for RNA polymerase binding has been well established for promoters of several bacterial organisms such as *Bacillus subtilis*, *C. tracomatis, E. coli*, and *Klebsiella pneumoniae *[23,51-56]. Previous reports also suggest that the inverted and direct repeats contribute to the DNA curvatures, thus affecting RNA polymerase binding to the -35 and -10 regions [23,39]. Although less likely, the presence of *E. coli *regulators that are homologues of *E. chaffeensis *may also bind and influence the promoter activity. For example, homologues of *R. prowazekii *repressors/enhancers in *E. coli *have been reported for the 16S rRNA gene [32]. Variations in the promoter activity of *E. chaffeensis *genes observed in *E. coli *for the deletion constructs may not represent what may occur in the pathogen. Defining the importance of the putative regulatory domains of p28-Omp genes identified in this study requires further analysis in *E. chaffeensis *or using *E. chaffeensis *RNA polymerase.

Deletion of the consensus -35 region alone or in combination with the -10 region, but not of the -10 region alone, reduced the promoter activity to background levels for both genes 14 and 19. These data suggest that, independent of the gene assessed, the -35 regions identified contribute to the RNA polymerase binding. It is unclear why deletions of the predicted -10 regions for both the genes had little effect in altering the promoter functions. Greater tolerance to the loss of the -10 regions compared to -35 regions is reported for other prokaryotes [26,57-59]. It is, however, possible that the -10 regions we predicted are not accurate and may be present at a different location. Alternatively, the -10 regions may be less important in *E. chaffeensis*. This hypothesis is too premature at this time; more detailed mapping of the -10 regions is needed.

In the absence of genetic manipulation methods, an *in vitro *transcription system can serve as a useful molecular tool for mapping the molecular basis for differences in *E. chaffeensis *gene expression. For example, extensive studies have already reported using *in vitro *transcription systems to map regulation of gene expression for another intra-phagosomal bacterium, *C. trachomatis*, for which genetic manipulation systems are yet to be established [28-30]. In the current study, we also presented the first evidence for a similar *in vitro *transcription protocol to drive expression from two *E. chaffeensis *promoter sequences. More detailed investigations may also be performed by using the *in vitro *transcription protocol with *E. coli *or *E. chaffeensis *RNA polymerase, similar to studies carried out for *C. trachomatis *and *R. prowazekii *[23-30,32].

## Conclusion

In this study, we performed detailed RNA analysis to demonstrate that *E. chaffeensis *regulates transcription by sensing differences in host cell environments. Experimental evidence presented in this study also demonstrates that gene expression differences are achieved by altering changes in RNA polymerase activity influenced by the sequences located upstream to the transcription start sites. More detailed investigations are needed to map the mechanisms controlling gene expression in *E. chaffeensis *in different host cell environments.

## Methods

### *In vitro *cultivation of *E. chaffeensis*

*E. chaffeensis *Arkansas isolate was cultured *in vitro *in the canine macrophage cell line (DH82) and in the tick cell line (ISE6) as described previously [1,60].

### Nucleic acids isolation

About 20 ml of 90–100% infected *E. chaffeensis *confluent monolayers of DH82 or ISE6 cell cultures recovered from a T-150 flask were used for isolation of total RNA. Total RNA was isolated with the Tri-reagent method by following the manufacturer's instructions (Sigma-Aldrich, St. Louis, MO). The RNA pellet recovered was resuspended in 100 μl of nuclease-free water containing 40 units of RNase inhibitor, RNasin, (Ambion Inc., Austin, TX) and stored at -70°C until use. Quality and concentration of RNA were assessed by spectrophotometry with an ND-1000 spectrophotometer (Nanodrop Technologies, Wilmington, DE) and by calculating the ratio between the optical densities at 260 nm and 280 nm.

Genomic DNA was isolated from 2 ml of 90–100% infected *E. chaffeensis *confluent monolayer by the sodium dodecyl sulfate (SDS), proteinase K, phenol, chloroform, isoamyl alcohol method [61]. Final purified DNA was resuspended in 100 μl of TE buffer (pH 8.0); concentration was assessed by spectrophotometry with an ND-1000 spectrophotometer and stored at -20°C. Quality of DNA and RNA samples was further confirmed by resolving about 1–5 μg each on a 0.9% agarose gel or 1.5% formaldehyde agarose gel, respectively [61].

### Oligonucleotides

Oligonucleotides used for the experiments described in this study are custom synthesized from Integrated DNA Technologies (Coralville, IA) and are listed in Table 1.

**Table 1:** List of oligonucleotides used for this study

Primers	Sequence	Orientation	Amplicon size (bp)	Annealing temperature (°C)
PROMOTER ANALYSIS				
**Gene 14-upstream sequence primers**				
**For cloning into pPROBE-NT**				
RRG 183*	5' GACTCTAGAttgctcaacccataaaataatg	Forward	596	50
RRG 184	5' AGTGAGCTCtttataaaagataataaaaatttaag	Reverse		
				
**For cloning into pBlue-TOPO**				
RRG 217	5' attgctcaaccataaaataatggga	Forward	581	48
RRG 218	5' gttaataaaccttttataaaag	Reverse		
				
RRG 267	5' cagttaactttctgtaaacttc	Forward	521	48
RRG 218**		Reverse		
				
RRG 268	5' atcataagtttacaataatgtc	Forward	461	48
RRG 218		Reverse		
				
RRG 269	5' cgttttctgctttattagaatg	Forward	400	48
RRG 218		Reverse		
				
RRG 270	5' gttccgtatttattaatatatg	Forward	350	48
RRG 218		Reverse		
				
RRG 271	5' catgtactgaatttgtgatttg	Forward	286	48
RRG 218		Reverse		
				
RRG 272	5' ggataagtactttagcaagtgg	Forward	222	48
RRG 218		Reverse		
				
RRG 273	5' taagtagtaaagttaactatag	Forward	169	48
RRG 218		Reverse		
				
RRG 274	5' acttttgttgtaaatttgaaag	Forward	105	48
RRG 218		Reverse		
				
RRG 217		Forward	516	50
IG14-35 del R	5' (PO_4_)-gtctagaatataaaatttctttc	Reverse		
				
IG14-10 del F	5' (PO_4_)-taaatttttattatcttttataaaaggtttattaac	Forward	8366	56
IG14-10 del R	5' (PO_4_)-atgaaagaaataaagaaaagcaagtctag	Reverse		
				
IG14-35 del F	5' (PO_4_)-ttctttatttctttcattattc	Forward	8366	48
IG14-35 del R		Reverse		
				
IG14-10 del F		Forward	8343	51
IG14-35 del R		Reverse		
				
**Gene 19-upstream sequence primers**				
**For cloning into pPROBE-NT**				
RRG 185	5' GACTCTAGActtttaattttattattgccacatg	Forward	334	61
RRG 186	5' AGTGAGCTCaatagtgacaaataaattaacaatag	Reverse		
				
**For cloning into pBlue-TOPO**				
RRG 185		Forward	308	60
RRG 445	5' atataacctaatagtgacaaataaattaac	Reverse		
				
RRG 275	5' gtggcaaaagaatgtagcaataag	Forward	239	50
RRG 445		Reverse		
				
RRG 276	5' gtgctgtttttctcacctttacac	Forward	188	63
RRG 445		Reverse		
				
RRG 277	5' ctgacgtaatatattaaattttcc	Forward	125	55
RRG 445		Reverse		
				
RRG 185		Forward	267	50
IG19-35 del R	5' (PO_4_)-gtcagaatataaatttttgtataaaatatcg	Reverse		
				
IG19-10 del F	5' (PO_4_)-taatttatttgtcactattaggttat	Forward	8112	56
IG19-10 del R	5' (PO_4_)-gtagaagtgtcatataaaagcaag	Reverse		
				
IG19-35 del F	5' (PO_4_)-ttatatgacacttctactattgttaatttatttg	Forward	8112	61.5
IG19-35 del R		Reverse		
				
IG19-10 del F		Forward	8088	58
IG19-35 del R		Reverse		
				
PRIMER EXTENSION ANALYSIS				
**Gene 14**				
RRG 14-5'rev	5' gccttctctgctgtcgttgattcc		NA	52
				
**Gene 19**				
RRG 20-PEXT	5' cgttaataccactacctgctgggtcg		NA	58
RRG 44	5' cgcttccgtcccaattttgcttc		NA	58
				
*IN VITRO *TRANSCRIPTION ASSAY				
**Gene 14 upstream full-length+lac Z segment**				
RRG 217	5' attgctcaaccataaaataatggga	Forward	882	50
RRG 226	5' cgccattcgccattag	Reverse		
				
RRG 218	5' gttaataaaccttttataaaag	Forward	882	50
RRG 226		Reverse		
				
**Gene 19 upstream full-length+lac Z segment**				
RRG 217	5' attgctcaaccataaaataatggga	Forward	601	50
RRG 226		Reverse		
				
RRG 445	5' atataacctaatagtgacaaataaattaac	Forward	601	50
RRG 226		Reverse		
				
*IN VITRO *TRANSCRIPTION COUPLED TRANSLATION ASSAY				
RRG 185	5' gactctagacttttaattttattattgccacatg	Forward	848	58
RRG 247	5' tccggctcgtatgttgtgtg	Reverse		

* Text in capital letters refers to sequences inserted for creating restriction enzyme sites. ** Primer sequences were presented only once when a primer was described for the first time.

### Primer extension analysis

Primer extension analysis was performed by using a Primer Extension System AMV Reverse Transcriptase kit (Promega, Madison, WI). Briefly, oligonucleotides complementary to the transcripts of p28-Omp genes 14 and 19 were end labeled with [γ-^32^p] ATP using T4 polynucleotide kinase (Promega, Madison, WI) at 37°C for 10 min. The kinase reaction was stopped by heat inactivation at 90°C for 2 min. The end labeled primers (one ρ mole each) were annealed to *E. chaffeensis *RNA (~10 μg) by incubating at 58°C for 20 min in 11 μl reactions containing AMV primer extension buffer. *E. chaffeensis *RNA used for this experiment was isolated from cultures when the infection reached to 80–100%. One micro liter of AMV reverse transcriptase (1 unit) was added, and the reaction was incubated at 42°C for 30 min. The reaction products were concentrated by ethanol precipitation and electrophorosed on a 6% polyacrylamide gel containing 7 M urea, and the gel was transferred to a Whatman paper, dried and exposed to an X-ray film. The primer extended products were detected after developing the film with a Konica film processor (Wayne, NJ).

### Quantitative RT-PCR analysis

Quantitative differences in transcripts for p28-Omp genes 14 and 19 were assessed with a TaqMan-based diplex RT-PCR assay using gene-specific primers and probes as we reported earlier [19]. The analysis was performed on total RNA isolated for *E. chaffeensis *infected DH82 and ISE6 cells at 12, 24, 48, 72, 96 and 120 h post infection. Quantitative data relative to the number of *Ehrlichia *organisms were calculated [9,19].

### Bioinformatics analysis

Sequences upstream from the protein coding regions of *E. chaffeensis *p28-Omp 14 and 19 were obtained from the GenBank data base and aligned by using the genetic computer group (GCG) programs PileUp and Pretty [62] to search for sequence homologies. Direct repeats and palindrome sequences in the upstream sequences were identified with the GCG programs Repeat and StemLoop, respectively. *E. coli *σ70 promoter consensus sequences (-10 and -35) [63] were used to locate similar elements manually in p28-Omp genes 14 and 19 sequences upstream to the transcription start sites.

### Promoter constructs

Promoter constructs for p28-Omp genes 14 and 19 were made with two independent promoterless reporter genes containing plasmid vectors pPROBE-NT [64] and pBlue-TOPO (Invitrogen Technologies, Carlsbad, CA). The pPROBE-NT vector contains a GFP gene as the reporter gene, whereas a lacZ gene is the reporter gene in the pBlue-TOPO vector. To generate a p28-Omp gene14 promoter region construct, the entire non-coding sequences located between coding sequences of p28-Omp genes 13 and 14 were amplified by using *E. chaffeensis *genomic DNA as a template and the sequence-specific oligonucleotides (Table 1). A similar strategy was used to prepare the gene 19 promoter constructs by amplifying the DNA segment located between the coding regions of p28-Omp genes 18 and 19. The PCR products were ligated into the promoterless pBlue-TOPO and pPROBE-NT vectors and transformed into *E. coli *strain, Top10 (Invitrogen Technologies, Carlsbad, CA) and DH5α strain, respectively [61]. One clone each in forward and reverse orientations was selected for the genes 14 and 19 in the pBlue-TOPO plasmid. For the pPROBE-NT constructs, only forward orientation inserts containing plasmids were selected. In addition, nonrecombinant plasmids transformed in *E. coli *were selected to serve as negative controls.

### Promoter deletion constructs

Various deletion fragments of the promoter regions lacking parts of the 5' or 3' end segments of genes 14 and 19 were also generated by PCR and cloning strategy in the pBlue-TOPO plasmid. Deletion constructs of gene 14 and 19 promoters that are lacking the predicted -35 or -10 alone or the regions spanning from -35 to -10 were also generated by PCR cloning strategy but by using a Phusion site-directed mutagenesis kit as per the manufacturer's recommendations (New England Biolabs, MA). Primers used for the deletion analysis experiments are included in Table 1. Presence of correct inserts for the clones was always verified by restriction enzyme and sequence analysis.

### Assessment of promoter activity *in vitro*

Promoter region and reporter gene segments were amplified by PCR using pBlue-TOPO promoter constructs as the templates. Amplicons were then used for *in vitro *transcription reactions. The entire gene 14 upstream, 5' end non-coding region in forward or reverse orientations along with a 301 bp lacZ gene fragment were amplified from the constructs in pBlue-TOPO (described previously). A similar strategy was followed to generate gene 19 promoter region templates for use in the *in vitro *transcription analysis. PCR products were purified with the QIAquick PCR Purification Kit (Quiagen, Valencia, CA).

*In vitro *transcription analysis was performed by following protocol described previously [65] with minor modifications. Briefly, assays were performed in a 10 μl reaction containing 50 mM Tris-acetate (pH 8.0), 50 mM potassium acetate, 8.1 mM magnesium acetate, 27 mM ammonium acetate, 2 mM dithiothreitol, 400 μM ATP, 400 μM GTP, 400 μM UTP, 1.2 μM CTP, 0.21 μM [α-^32^P] CTP, 18 U of RNasin, 5% glycerol, 100 ng of purified PCR templates and 0.03 U of *E. coli *RNA polymerase holoenzyme (Epicentre, Madison, WI). The reaction was incubated at 37°C for 15 min and then terminated by adding 4 μl of stop solution (95% formamide, 20 mM EDTA, 0.05% bromophenol blue, 0.05% xylene cyanol). Four micro liters of reaction contents each were resolved in a 6% polyacrylamide gel containing 7 M urea [66]. The gel was transferred to a Whatman paper, dried and exposed to an X-ray film; the *in vitro *transcripts were detected after developing the film with a Konica film processor (Wayne, NJ).

### Assessment of promoter activity in *E. coli*

The pPROBE-NT constructs containing promoter regions of genes 14 and 19 were assessed for promoter activities by observing green florescence emitted from colonies on agar plates. The promoter activity was further confirmed by performing Western blot analysis using a GFP polyclonal antibody (Rockland Immunochemicals, Inc., Gilbertsville, PA) on protein extracts made from *E. coli *containing the recombinant plasmids. The pBlue-TOPO promoter constructs were also evaluated for promoter activity by measuring β-galactosidase activity. To accomplish this, *E. coli *colonies containing the recombinant plasmids were grown to an optical density of 0.4 (at 600 nm); soluble protein preparations from the cell lysates were prepared and assessed for the lacZ expression by using a β-gal assay kit as per the manufacturer's instructions (Invitrogen Technologies, Carlsbad, CA,). About 2.5 or 5 μg of protein preparations were assessed for the β-galactosidase activity using Ortho-Nitrophenyl-β-D-Galactopyranoside (ONPG) as the substrate. The analysis included protein preparations made from no-insert controls as well as *E. coli *cultures containing constructs with promoter segments in the reverse orientation. The experiments were repeated four independent times with independently isolated protein preparations; samples were also assayed in triplicate each time. Specific activity of β-galactosidase was calculated using the formula outlined in the β-gal assay kit protocol.

### Statistical Analysis

Statistical analysis of RT-PCR experiments for measuring the quantitative differences in the gene expression of p28-Omp genes14 and 19 was performed by using the unpaired Student *t*-test. For promoter deletion analysis experiments, statistical analysis was performed by using repeated measures of ANOVA, and the Bonferroni method was used to adjust for multiple comparisons. GraphPad InStat Software (La Jolla, CA) was used to perform these analyses. A *P *value of less than 0.05 was considered significant.

## Authors' contributions

LP carried out the RNA mapping studies, promoter deletion analysis, in vitro transcription experiments, statistical analysis, and also drafted the manuscript. CC carried out the cell culture experiments, participated in *in vitro *transcription experiments and compiling references and manuscript editing. RRG conceived of the study and participated in its design and coordination, was instrumental in obtaining financial support, and helped in data analysis and drafting the manuscript to its final form. All authors read and approved the final manuscript.
